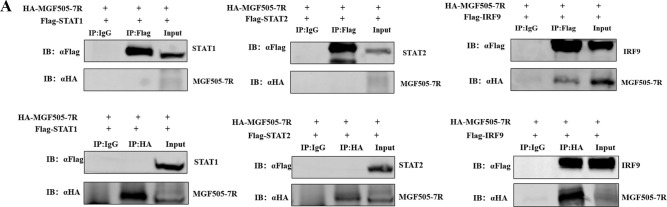# Correction for Huang et al., “African Swine Fever Virus MGF505-7R Interacts with Interferon Regulatory Factor 9 to Evade the Type I Interferon Signaling Pathway and Promote Viral Replication”

**DOI:** 10.1128/jvi.00249-26

**Published:** 2026-03-26

**Authors:** Zhao Huang, Haoxuan Cao, Fanliang Zeng, Sizhan Lin, Jianglin Chen, Yi Luo, Jianyi You, Cuiying Kong, Zhanzhuo Mai, Jie Deng, Weiting Guo, Xiongnan Chen, Heng Wang, Pei Zhou, Guihong Zhang, Lang Gong

## AUTHOR CORRECTION

Volume 97, no. 3, e01977-22, 2023, https://doi.org/10.1128/jvi.01977-22. Figure 3A should appear as shown in this correction. Due to an error during figure assembly, the Western blot image in panel A was placed incorrectly. The Co-IP results of exogenous STAT1 with MGF505-7R and STAT2 with MGF505-7R have been corrected. The results and conclusions of the study remain unaffected. We sincerely apologize for this error.

**Fig 3:**